# Integrative TCM Conservative Therapy for Low Back Pain due to Lumbar Disc Herniation: A Randomized Controlled Clinical Trial

**DOI:** 10.1155/2013/309831

**Published:** 2013-06-24

**Authors:** Wei An Yuan, Shi Rong Huang, Kai Guo, Wu Quan Sun, Xiao Bing Xi, Ming Cai Zhang, Ling Jun Kong, Hua Lu, Hong Sheng Zhan, Ying Wu Cheng

**Affiliations:** ^1^Shuguang Hospital Affiliated to Shanghai University of Traditional Chinese Medicine, Shanghai 201203, China; ^2^Yueyang Integrative Traditional Chinese and Western Medicine Hospital Affiliated to Shanghai University of Traditional Chinese Medicine, Shanghai 200437, China; ^3^Ruijin Hospital Affiliated to Shanghai Jiaotong University, Shanghai 200025, China

## Abstract

Low back pain due to lumbar disc herniation (LDH) is very common in clinic. This randomized controlled trial was designed to investigate the effects of integrative TCM conservative therapy for low back pain due to LDH. A total of 408 patients with low back pain due to LDH were randomly assigned to an experimental group with integrative TCM therapy and a control group with normal conservative treatment by the ratio of 3 : 1. The primary outcome was the pain by the visual analogue scale (VAS). The secondary outcome was the low back functional activities by Chinese Short Form Oswestry Disability Index (C-SFODI). Immediately after treatment, patients in the experimental group experienced significant improvements in VAS and C-SFODI compared with the control group (between-group difference in mean change from baseline, −16.62 points, *P* < 0.001 in VAS; −15.55 points, *P* < 0.001 in C-SFODI). The difference remained at one-month followup, but it is only significant in C-SFODI at six-month followup (−7.68 points, *P* < 0.001). No serious adverse events were observed. These findings suggest that integrative TCM therapy may be a beneficial complementary and alternative therapy for patients with low back pain due to LDH.

## 1. Introduction

Lumbar disc herniation (LDH) is a common disease and a major contributing factor of low back pain [1, 2]. Although many studies have confirmed that surgery is more effective for LDH [3–5], conservative therapies have also been recognized for their therapeutic efficacy [3, 4]. Considering the fact that 20% of patients still have pain after surgery [6], 7% to 15% of surgical patients may have failed back surgery syndrome [7], and some patients are scared of surgery, conservative treatment is still one of the primary means for LDH.

In China, TCM is one of the main conservative treatments for LDH. Previous studies have confirmed that some TCM therapies have certain effects on low back pain due to LDH. These include acupuncture [8, 9], oral administration of Chinese medicine [10], external application of Chinese medicine [11], Chinese Tuina (massage) [12], and TCM-characteristic functional exercise [13]. Clinically, these therapeutic methods are not used alone but often in combination [12]. Recently, the clinical pathway of treating LDH with integrative TCM therapy has attracted attention [14]. The Shi's Traumatology Medical Center of Shuguang Hospital Affiliated to Shanghai University of Traditional Chinese Medicine is well recognized for its long-term commitment to the research on conservative treatment for LDH, coupled with a package protocol for LDH. However, high-quality research evidence is needed to support the effectiveness of the protocol.

This clinical trial aims to study the efficacy and safety of integrative TCM therapy for LDH and thus confirm its clinical effect. 

## 2. Materials and Methods

### 2.1. Design

We conducted a multicenter, randomized controlled trial to evaluate the effectiveness of integrative TCM conservative treatment for patients with low back pain due to LDH. Patients were randomly assigned to an experimental group and a control group by the ratio of 3 : 1 using computer-generated numbers. The randomized treatment assignments were sealed in opaque envelopes and opened individually for each patient who agreed to be in the study. The nurse, who had no role in the design and conduct of the study, prepared the envelopes. Patients in the experimental group were treated with integrative TCM therapy once a day, for two weeks, whereas patients in the control group were treated with a two-week normal conservative intervention. At baseline, immediately after treatment, one and six months after treatment, visual analogue scale (VAS) and the Chinese Short Form Oswestry Disability Index (C-SFODI) were used as outcome assessment. This trial is registered in Chinese Clinical Trial Registry (No. ChiCTR-TRC-11001343).

### 2.2. Subjects

Patients were recruited from Shuguang Hospital Affiliated to Shanghai University of Traditional Chinese Medicine, Ruijin Hospital Affiliated to Shanghai Jiaotong University, and Yueyang Integrative Traditional Chinese and Western Medicine Hospital Affiliated to Shanghai University of Traditional Chinese Medicine between January 2011 and August 2012.

Inclusion criteria: (1) aging 20–60 years; (2) having low back pain due to LDH (MRI scan confirmed lumbar disk herniation) and ruling out other relevant ongoing pathologies such as fractures, lumbar spondylolisthesis, tumor, osteoporosis, or infection; (3) willing to participate in this study and signing the informed consent.

Exclusion criteria: (1) having other pain syndromes; (2) experiencing a history of spinal surgery; (3) having neurological disease; (4) having psychiatric disease; (5) having serious chronic diseases that could interfere with the outcomes (e.g., cardiovascular disease, rheumatoid arthritis, epilepsy, or other disqualifying conditions); (6) scared of acupuncture; (7) pregnant or planning to become pregnant during the study; (8) having other diseases that the researchers believe is not suitable for the study.

### 2.3. Treatment

#### 2.3.1. Experimental Group

Patients in the experimental group receive a two-week integrative TCM treatment. They were further divided into three subgroups (according to the duration from initial low back pain to getting treatment) for different treatment methods: acute stage (0–14 days), subacute stage (15–30 days), and chronic stage (>30 days).

*Acute stage: *(1) Electroacupuncture + (2) Chinese herbal injection (Salvia miltiorrhiza injection) + (3) external plaster (Compound Redbud Injury-healing Cataplasms); *Subacute stag:* (1) Chinese Tuina (massage) + (2) hot compress using Chinese medicine + (3) external plaster (Compound Redbud Injury-healing Cataplasms); *Chronic stage: *(1) TCM functional exercise + (2) external plaster (Compound Redbud Injury-healing Cataplasms). 

*Treatment Parameters*

*Electroacupuncture.* Points: bilateral Dachangshu (BL 25) and Baihuanshu (BL 30).

Method: Insert the needles (the sterile, disposable needles, 0.3 × 75 mm, manufactured by Suzhou Medical Supplies Factory Co., Ltd.) 2.5 to 2.8 cun. Upon De Qi (needling sensation), connect the needles with the electroacupuncture device (Model: G6805-II, manufactured by Guangzhou KangMai Medical Devices Co., Ltd.), using a continuous wave, an electrical stimulation pulse wave of approximately 0.6 ms and a frequency of 20 Hz. The treatment was conducted once every day, 30 min for each treatment.

*External Plaster.* Compound Redbud Injury-healing Cataplasms (Approval no. Z19991106, manufactured by Shanghai LEY's Pharmaceutical Co., Ltd.).

Main ingredients: Zi Jing Pi (*Cortex Cercis Chinensis)*, Huang Jing Zi (*Negundo Chastetree Fruit*), Da Huang (*Radix et Rhizoma Rhei*), Chuan Xiong (*Rhizoma Chuanxiong*), Tian Nan Xing (*Rhizoma Arisaematis*), and Ma Qian Zi (*Semen Strychni*).

Functions: Circulates blood, resolves stasis, eliminates swelling, and alleviates pain.

Method: Apply the cataplasms to the most painful area, one plaster each time, once a day.

*Chinese Herbal Injection.* Salvia miltiorrhiza injection (Approval no. Z51021303, manufactured by Sichuan ShengHe Pharmaceutical Co., Ltd.).

The main ingredient of the injection is Salvia root P.E. It acts to circulate blood and resolve stasis.

Method: Intravenous dripping of 20 mL salvia miltiorrhiza injection and 250 mL 5% glucose, once a day.

*Hot Compress Using Chinese Medicine.* Ingredients: 20 g of Cang Zhu (*Rhizoma Atractylodis*), Qin Jiao (*Radix Gentianae Macrophyllae*), Sang Zhi (*Ramulus Mori*), Mu Gua (*Fructus Chaenomelis*), Hong Hua (*Flos Carthami*), Chuan Xiong (*Rhizoma Chuanxiong*), Hai Feng Teng (*Caulis Piperis Kadsurae*) and Lei Gong Teng (*Radix Tripterygii Wilfordii*), respectively. All herbs were provided by Shanghai Hongqiao Pharmaceutical Co., Ltd. and have been tested and qualified.

Method: Place the previous medicinal into a gauze bag, decoct with water for 20 mins and take it out. After the temperature cooled to 40~45°C, apply the back to the affected low back area for 30–40 minutes, once a day. The hot compress can help circulate blood and resolve stasis.

*TCM Functional Exercise.* The exercise is known as “Fei Yan Shi” (literally meaning *“the flying swallow style*”) in Chinese.

Method: Ask the patient to take a prone position, extend both hands backwards, lift the chest and lower limbs off the bed using the abdomen as a pivot, and then relax. Conduct this exercise once a day and repeat 4-5 times each time. 

Functions: Strengthens the power of back muscles, increases the stability of the spine, and thus prevents relapses.

*Chinese Tuina (Massage).* Ask the patient to take a prone position and find the tenderness spots on the low back. Then apply *gun*-rolling (10 min), *Anrou*-pressing and kneading (10 min), and *Tanbo*-plucking (5 min) manipulation to the tenderness spots and surrounding areas. Conclude with oblique pulling manipulation of the low back. Conduct the treatment once a day.

Functions: Relaxes spasm of the low back muscles and adjusts lumbar subluxation.

After one week TCM treatment, if the patient's lower back pain without any relief or even aggravated, the prescription of pain medication was adjusted according to clinical guidelines [15], detailed records the type and dose of pain medication taken by patients, and the patient was identified as no effect.

#### 2.3.2. Control Group

Patients in the control group receive a two-week normal conservative treatment [3]. Intervention measures include three sections, (1) health education. The patients were invited to receive LDH health education twice a week in outpatient; the health education was designed exclusively to inform patients about the natural course of their illness and the expectation of successful recovery, irrespective of the initial intensity of their pain, educate patients to avoid some bad habits that aggravate the disease, such as a sitting position for a long time and carrying heavy loads, and encourage patients to participate in social activities. (2) Rest: in addition to the normal sleep, the patients need to rest in bed for at least 1-2 hours a day. (3) Pain medication or physical therapy: after one week health education, if the patient's lower back pain without any relief or even aggravated, the prescription of pain medication was adjusted according to clinical guidelines [15], detailed records the type and dose of pain medication taken by patients. And if the patients do not want to take pain medication, then the patients were referred to a physiotherapist.

### 2.4. Measurements

All outcomes were assessed by observers unaware of the grouping, at baseline (M1), immediately after the last intervention (M2). The followup included the assessments at one month (M3) and six months (M4) after the last intervention.

The primary outcome measure was the change in pain by the visual analogue scale (VAS), scores range 0 to 100, and a higher score indicates a greater pain, 0 means no pain, and 100 means intolerable pain.

The secondary outcome measure was the change in the Chinese Short Form Oswestry Disability Index (C-SFODI), range 0 to 100%. The C-SFODI consists of nine questions, which come from Oswestry Disability Index (ODI); omit the sex life question in Section  8, because this question is always unacceptable by Chinese. The C-SFODI calculation formula is actual cumulative score/45 × 100%, with higher percentage indicating more severe functional disability. And the study has shown that the C-SFODI has good reliability and validity [16].

### 2.5. Statistical Analysis

Our pretrial power calculation indicated that 81 patients in experimental group were required to detect a difference in pain relief based on the preliminary experiment data at a significant level of 5% (a two-sided *t*-test) with 80% power. In anticipation of a 20% attrition rate, we sought 102 patients at least in experimental group. Taking into account the poor effect of control therapy, 102 patients were included in the control group.

Between-group difference at baseline was analyzed using independent-samples *t*-test or Chi-square test. Changes in continuous measures were analyzed by analysis of variance (ANOVA). Effects were evaluated on an intention-to-treat basis (ITT), and participants who did not complete the followup period were considered not having any changes in scores. A two-sided *P* value of less than 0.05 indicated statistical significance. Results are presented as mean and standard deviation (SD) at M1 and as between-group difference with 95% confidence intervals (CI) at M2, M3, and M4.

### 2.6. Quality Control

Before the beginning of the study, all researchers have to receive protocol training. A clinic research coordinator (CRC) was employed to assist researchers in each center. A monitor was also appointed to ensure the quality of the research.

## 3. Results

**Figure 1:**
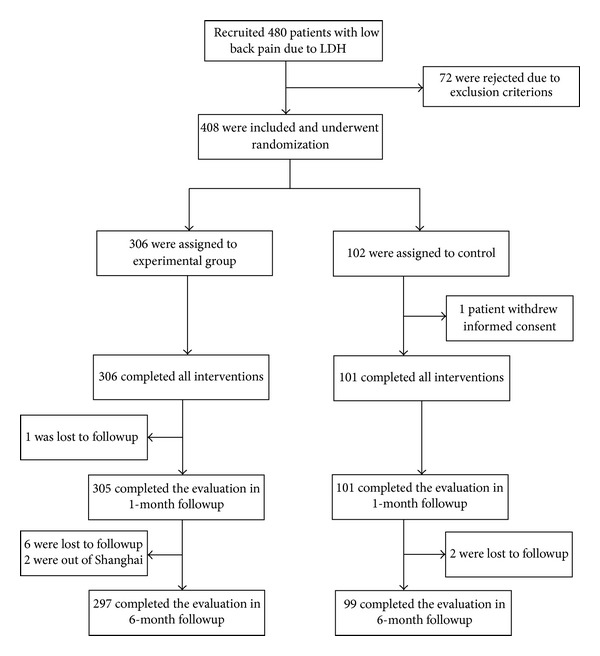
Screening, randomization, and completion evaluations from the baseline to six-month followup, LDH = lumbar disc herniation.

Between January 2011 and August 2012, a total of 480 patients with low back pain due to LDH were recruited, 72 were rejected due to exclusion criterions, and 408 eligible patients were randomly assigned in accordance with the ratio of 3 : 1 to the experimental group and the control group, 306 in the experimental group and 102 in the control group. Patients in the experimental group all completed a two-week treatment. In the control group, at the second week one patient in the control group was unwilling to continue to participate and withdrew his informed consent, and two patients took Fenbid (500 mg for each dose, 2 doses a day) since the pain worsened during treatment (Figure 1).

### 3.1. Baseline Characteristics of the Patients

Table 1 shows the baseline data for the 408 participants. The mean age of all patients is 45 years, and 51% were women. In terms of disease staging, experimental group and control group were comparable. And the baseline outcome including VAS scores and C-SFODI were also reasonably well balanced between experimental group and control group.

### 3.2. Improvement in the Primary Outcome

**Figure 2:**
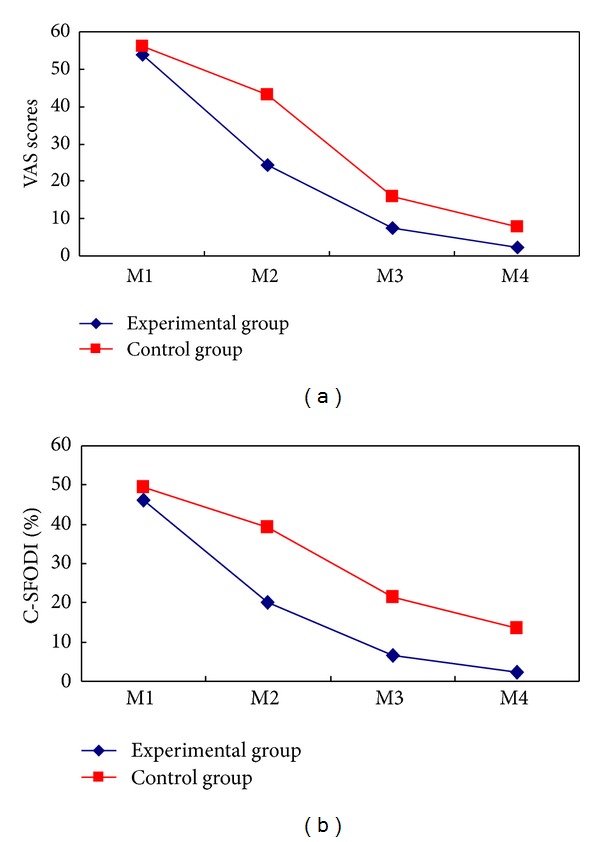
Mean changes of the primary and secondary outcomes. The means of outcomes are shown for the experimental group (diamond) and the control group (squares). Measurements were obtained at baseline (M1), immediately after the last intervention (M2). The followup included the assessments at one month (M3) and six months (M4) after the last intervention. Visual analogue scale (VAS, scores rang 0 to 100) with higher scores indicating greater pain. The Chinese Short Form Oswestry Disability Index (C-SFODI, range 0 to 100%) consists of 9 questions, with higher percentage indicating more severe functional disability.

The changes in the primary outcomes from baseline to six-month followup are shown in Table 2 and Figure 2. Immediately after the intervention, two groups showed significant decrease in VAS than the baseline. And the experimental group showed a more significant decrease than the control group (−16.62 points [95% confidence interval {CI}, −20.25 to −12.98]; *P* < 0.001).

One month after intervention, two groups also had significantly greater reduction in VAS than the baseline. And again, the experimental group showed a more significant decrease than the control group (−6.37 points [95% CI, −10.20 to −2.54]; *P* = 0.001).

Six months after intervention, compared with the baseline, the changes in VAS remained significant in the experimental group and control group, but between-group difference was not significant (*P* = 0.091).

### 3.3. Improvement in the Secondary Outcome

Immediately after intervention, two groups had significant improvement in C-SFODI than the baseline, and the experimental group showed a more significant improvement than the control group (−15.55 points [95% CI, −18.92 to −12.18]; *P* < 0.001).

One month after intervention, two groups also had significant improvement in C-SFODI than the baseline. And again, the experimental group improved more (−11.37 points [95% CI, −14.62 to −8.11]; *P* < 0.001).

Six months after intervention, two groups also maintained significant improvement, and the experimental group showed superiority (−7.68 points [95% CI, −11.42 to −3.94]; *P* < 0.001). 

### 3.4. Adverse Events

One patient in the experiment group had mild fainting during acupuncture, remission by bed rest, and then completed the remaining treatment. Two patients in the control group were given Fenbid orally due to aggravated low back pain. No other adverse events were noted in either experimental group or control group.

## 4. Discussion

Although the mechanism of low back pain caused by lumbar disc herniation (LDH) is still not very clear, the prevailing view is that low back pain due to LDH was found to occur not only in response to mechanical stimuli but also to chemical irritation around the nerve root sheath and sinuvertebral nerve [17].

Different TCM therapies have different advantages in the treatment of LDH. Pain is the main symptom in the acute stage of LDH; acupuncture has good analgesic effect on low back pain due to LDH [8]. Lumbar dysfunction is the main symptom in the remission stage; Chinese massage has good effect on improving dysfunction [12]. Oral Chinese herbal formulae [10], external use of Chinese medicine [11], and Chinese herbal injection [18] also showed good effect in relieving pain and improving dysfunction caused by LDH. And one study also found that Salvia miltiorrhiza injection especially works better and faster for the acute stage when compared with mannitol [19]. Although the mechanism of acupuncture, Chinese massage, and traditional Chinese herbs in the treatment of LDH remains unclear, it is generally agreed that these treatment methods play a role by increasing local blood circulation, relieving nerve root edema, and speeding up the metabolism of the local inflammatory mediators. In recovery stage of the disease, the major task is to strengthen the muscles of the waist and abdomen to prevent relapse [20], and TCM functional exercise has advantages in this regard and can subsequently increase the lumbar stability to prevent recurrence [13].

Treating LDH according to different stages has been more and more accepted. In China, LDH is mainly divided into three stages, including acute stage, subacute stage (or remission stage), and chronic stage (or recovery stage) [21, 22]. Studies have proven that treating LDH according to different stages has obtained a good clinical effect [23]. In addition, studies have also suggested that it can obtain a better effect than treatment without differentiating different stages [24]. 

The past 20 years of clinical practice have witnessed the safety of the treatment regimens used in this study. At the same time, its efficacy has been preliminarily confirmed; however, high quality research evidence is still needed. In the treatment regimens, different TCM therapies were selected according to the characteristics of different stages. Specifically, acupuncture and Chinese herbal injections were used in the acute stage for fast pain relief, Chinese Tuina (massage) and external application of Chinese medicine were used in the subacute stage for improvement of the lumbar functions, and low back muscle exercise was used in the chronic stage to increase the stability of the spine and prevent relapses.

In China, nonsurgical treatment of lumbar disc herniation mainly uses drugs, physical therapy, or TCM treatment. TCM treatment used in the experimental group has been used in clinical routine and is considered to have good clinical efficacy; the efficacy of conservative treatment used in the control group is considered very weak, usually as auxiliary treatment of other therapies. Ethics Committee considers that in order to maximize the protection of the interests of the patients, it is necessary to let the patients have more opportunity to receive TCM treatment, so in this research the sample size of the experimental group and the control group is 3 : 1.

The findings of this study have shown that immediately and one month after intervention, integrative TCM conservative treatment can significantly reduce the VAS scores and C-SFODI, and at six month after intervention, integrative TCM conservative treatment can also significantly reduce the C-SFODI, but two groups have no significant difference in reducing VAS score. VAS is an international general pain visual analog scale, and C-SFODI is the improved version of the ODI (Oswestry Disability Index), and it consists of 9 questions, a higher percentage indicating a more severe functional disability.

Regarding adverse events, one patient had mild fainting in the experiment group, two patients in the control group were given Fenbid oral due to low back pain aggravation, and no other adverse events were noted in either experimental group or control group. The mechanism of integrative TCM conservative treatment for LDH remains unclear, and it will be our future research orientation.

The main limitation of this study is the short followup time. As a result, we failed to conduct comprehensive evaluation regarding the long-term efficacy of integrative TCM conservative treatment for LDH.

## 5. Conclusions

This randomized controlled clinical trial provides reliable evidence regarding the effectiveness of integrative TCM conservative treatment for patients with low back pain due to lumbar disc herniation. A large sample of long-term followup is further needed for future research.

## Figures and Tables

**Table 1:** Baseline characteristics of the study participants*.

Variable	Experimental group (*N* = 306)	Control group (*N* = 102)
Sex: no. of patients (%)		
Male (%)	147 (48.0)	53 (52.0)
Female (%)	159 (52.0)	49 (48.0)
Age (years)	45.91 ± 10.73	43.58 ± 12.10
Stage of the disease^§^: no. of patients (%)		
Acute stage (0~14 days)	109 (35.6)	38 (37.3)
Subacute stage (15~30 days)	101 (33.0)	32 (31.4)
Chronic stage (>30 days)	96 (31.4)	32 (31.4)
Segments of lumbar disc herniation^&^: no. of patients (%)		
One segment (L3/L4 or L4/L5 or L5/S1)	61 (19.9)	19 (18.6)
Two segments (L3/L4 and L4/L5 or L4/L5 and L5/S1)	196 (64.1)	67 (65.7)
Three segments (L3/L4 and L4/L5 and L5/S1)	49 (16.0)	16 (15.7)
VAS scores^#^ (0~100)	53.94 ± 19.60	56.00 ± 19.61
C-SFODI^†^ (0~100%)	46.07 ± 20.56	49.59 ± 22.53

*Plus-minus values are means ± SD unless otherwise noted.

^§^Stage of the disease is divided according to duration of the low back pain symptoms first appeared of this time, acute stage (0~14 days), subacute stage (15~30 days), and chronic stage (>30 days).

^#^Visual analogue scale (VAS, range 0 to 100) with higher scores indicating greater pain.

^&^Based on imaging examination to determine the segments of lumbar disc herniation; L3/L4 means disc herniation between the third and fourth lumbar, and the others are the same.

^†^The Chinese Short Form Oswestry Disability Index (C-SFODI, range 0 to 100%) consists of 9 questions. A higher percentage indicates a more severe functional disability.

**Table 2:** Changes in primary and secondary outcomes*.

Variable	Outcomes of different timepoints (means ± SD)^*▾*^		Mean change from baseline (95% CI)		Between-group difference (95% CI)	*P* value^§^
	Experimental group (*N* = 306)	Control group (*N* = 102)	Experimental group (*N* = 306)	Control group (*N* = 102)	Experimental group versus control group	
VAS scores^*※*^						
M1	53.94 ± 19.60	56.00 ± 19.61	—	—	—	0.358
M2	24.48 ± 17.73	43.16 ± 20.75	−29.46 (−31.52 to −27.40)	−12.84 (−15.58 to −9.83)	−16.62 (−20.25 to −12.98)	<0.001
M3	7.40 ± 6.52	15.83 ± 11.61	−46.54 (−48.50 to −44.57)	−40.17 (−43.26 to −37.07)	−6.37 (−10.20 to −2.54)	0.001
M4	2.32 ± 2.29	7.94 ± 6.76	−51.62 (−53.73 to −49.51)	−48.06 (−51.45 to −44.67)	−3.56 (−7.69 to 0.57)	0.091
C-SFODI^†^						
M1	46.07 ± 20.56	49.59 ± 22.53	—	—	—	0.145
M2	20.20 ± 13.79	39.26 ± 22.84	−25.88 (−27.70 to −24.05)	−10.33 (−13.17 to −7.48)	−15.55 (−18.92 to −12.18)	<0.001
M3	6.69 ± 5.98	21.57 ± 15.35	−39.38 (−41.33 to −37.44)	−28.02 (−30.64 to −25.39)	−11.37 (−14.62 to −8.11)	<0.001
M4	2.16 ± 2.01	13.36 ± 10.64	−43.91 (−46.07 to −41.75)	−36.23 (−39.30 to −33.16)	−7.68 (−11.42 to −3.94)	<0.001

*Values are means with means ± SD or the 95% confidence (CI). M1: measurements were obtained at baseline; M2: immediately after the last intervention; M3: one month after the last intervention; M4: six months after the last intervention.

^*▾*^The VAS scores means at different timepoints between two groups were analyzed by repeated measures analysis of variance. Within-subjects effects tests of different timepoints means, *F* = 1381.914, *P* < 0.001. Between-subjects effects tests of different group means, *F* = 46.322, *P* < 0.001. The C-SFODI means at different timepoints between two groups were analyzed by repeated measures analysis of variance. Within-subjects effects tests of different timepoints means, *F* = 1076.327, *P* < 0.001. Between-subjects effects tests of different group means, *F* = 78.879, *P* < 0.001.

^§^ *P* values were calculated with independent-samples *t*-test for mean change from baseline between two groups.

^*※*^Visual analogue scale (VAS, scores range 0 to 100) with higher scores indicating greater pain.

^†^The Chinese Short Form Oswestry Disability Index (C-SFODI, range 0 to 100%) consists of 9 questions, with higher percentage indicating more severe functional disability.
